# Acquisition of Freezing Tolerance in *Vaccinium macrocarpon* Ait. Is a Multi-Factor Process Involving the Presence of an Ice Barrier at the Bud Base

**DOI:** 10.3389/fpls.2022.891488

**Published:** 2022-05-04

**Authors:** Camilo Villouta, Beth Ann Workmaster, David P. Livingston, Amaya Atucha

**Affiliations:** ^1^Arnold Arboretum of Harvard University, Boston, MA, United States; ^2^Department of Horticulture, University of Wisconsin-Madison, Madison, WI, United States; ^3^Department of Crop and Soil Sciences, USDA-ARS and North Carolina State University, Raleigh, NC, United States

**Keywords:** bud anatomy, cold acclimation, cold hardiness, freeze dehydration, cranberry (*Vaccinium macrocarpon* Ait), fruit crop, ice propagation

## Abstract

Bud freezing survival strategies have in common the presence of an ice barrier that impedes the propagation of lethally damaging ice from the stem into the internal structures of buds. Despite ice barriers’ essential role in buds freezing stress survival, the nature of ice barriers in woody plants is not well understood. High-definition thermal recordings of *Vaccinium macrocarpon* Ait. buds explored the presence of an ice barrier at the bud base in September, January, and May. Light and confocal microscopy were used to evaluate the ice barrier region anatomy and cell wall composition related to their freezing tolerance. Buds had a temporal ice barrier at the bud base in September and January, although buds were only freezing tolerant in January. Lack of functionality of vascular tissues may contribute to the impedance of ice propagation. Pith tissue at the bud base had comparatively high levels of de-methyl-esterified homogalacturonan (HG), which may also block ice propagation. By May, the ice barrier was absent, xylogenesis had resumed, and de-methyl-esterified HG reached its lowest levels, translating into a loss of freezing tolerance. The structural components of the barrier had a constitutive nature, resulting in an asynchronous development of freezing tolerance between anatomical and metabolic adaptations.

## Introduction

Freezing temperatures are one most critical factors limiting woody plant geographical distribution. As buds sustain the development of new organs for the next growing season, their freezing survival mechanisms play a key role determining the low temperature threshold that each species can withstand (George, 1974; Levitt, 1980; George et al., 1982). The current increase in duration and frequency of extreme weather events due to climate change (Vasseur et al., 2014; Williams et al., 2015) increases the susceptibility to freezing damage of woody species, potentially altering their current geographical distribution (Gu et al., 2008). In the case of spring temperatures and frost occurrences, these changes can modify the phenology of plants, possibly causing an increased risk of bud damage by freezing temperatures (Inouye, 2000; Augspurger, 2013). Bud formation and reproductive tissue development occurs typically at the end of summer or in fall (Burke et al., 1976), thus winter survival of woody perennial species buds is crucial for their reproductive success. Investigating the internal changes that these structures undergo is necessary to understand the consequences of climate change for woody species (Bertrand and Castonguay, 2003).

Woody plant species have developed multiple strategies for the survival of buds during freezing temperatures, namely deep supercooling and freeze-induced dehydration (extraorgan freezing). While deep supercooling and freeze dehydration have some mechanistic differences, they do have in common the presence of a barrier to the propagation of ice located at the base of the bud (Levitt, 1980; Ishikawa and Sakai, 1985). This barrier impedes the propagation of lethally damaging ice originating from the stem toward the bud. While both strategies also share the common mechanism of extracellular ice formation tolerance in bud scales, how the ice barrier contributes to inner bud tissues’ survival differs (Pearce, 2001; Wisniewski et al., 2014). In deep supercooling, an ice barrier consisting of physical or structural anatomical modifications at the bud axis or bud base prevents ice nucleation in florets and meristems by sequestering small amounts of liquid water (Quamme, 1974; Andrews and Proebsting, 1986). When the critical nucleating temperature of this sequestered water is reached, ice propagation is rapid, and cellular damage is lethal (Quamme et al., 1995). In freeze-induced dehydration, the ice barrier stops ice propagation from the stem into the zone containing the meristems, and, along with independent extracellular ice formation in tolerant tissues, such as the bud scales, gradual dehydration of the inner bud tissues occurs. This dehydration is driven by the vapor pressure deficit established by the extracellular ice in the bud scales and is enabled by the presence of the ice barrier (Sakai, 1979; Ishikawa and Sakai, 1981, 1985; Ishikawa, 1982; Flinn and Ashworth, 1994; Endoh et al., 2014).

Several studies have reported the existence of ice barriers in multiple species (Ishikawa and Sakai, 1981; Ashworth, 1984; Ashworth et al., 1992; Stone et al., 1993; Kuprian et al., 2016), and they highlight a range of adaptations from vascular connection loss, changes in cell wall composition, or the presence and size of intercellular spaces. In the case of *Prunus* spp. buds, cells near the primordium procambium do not complete their differentiation into vessel elements in the fall, thus preventing ice propagation to the inner bud structures, further allowing the sequestration of supercooled water inside the bud (Ashworth, 1982, 1984; Ashworth et al., 1989; Callan, 1990; Kader and Proebsting, 1992). However, in spring, xylem differentiation resumes, resulting in the loss of supercooling capacity. Changes in the conformation of pectins, specifically homogalacturonan (HG) in the cell wall, have been associated with the development of freezing tolerance (Mohnen, 2008). HG is initially synthesized in a methyl-esterified form, which can be either totally or partially de-methyl-esterified (Knox et al., 1990). This process can increase rigidity and reduce porosity of cell walls, drastically reducing the movement of water molecules through them (Wisniewski et al., 1991; Rajashekar and Lafta, 1996; Lee et al., 2017), thereby acting as a barrier for ice propagation. In the case of intercellular spaces, their small size or absence has been described as critical to the prevention of ice propagation into inner bud tissues (Ashworth, 1984; Quamme et al., 1995; Jones et al., 2000).

*Vaccinium macrocarpon* Ait. is a woody perennial vine of commercial and ecological importance. Growing in cultivated sunken production beds and naturally in lowlands and marshes, it is frequently exposed to potentially injurious freezing temperatures. Diverse studies on this species have focused on the metabolic changes associated with the development of freezing tolerance (Palta et al., 1993; Ndlovu, 2015). However, contributions of anatomical modifications to the plant’s survival to freezing temperatures remain to be elucidated. During acclimation and the development of cold hardiness, *V. macrocarpon* experiences changes at the metabolic level; there is not only an increase in total nonstructural carbohydrates, as well as sucrose, fructose, and glucose, but there is also an increase in galactolipids (Ndlovu, 2015).

*Vaccinium macrocarpon* terminal buds survive freezing temperatures due to a process of freeze dehydration (Villouta et al., 2020), a process that includes the assumption of an ice barrier. Similarly, Workmaster and Palta (2006) suggested that the patterns of freezing damage observed in terminal buds after controlled freezing tests could be explained by the presence of an ice barrier. Although different techniques have been used to detect ice barriers, thermal videography techniques have been the most widely used (Hacker and Neuner, 2007, 2008; Hacker et al., 2008; Kuprian et al., 2014; Neuner and Kuprian, 2014). The first thermographic video recordings of *V. macrocarpon* shoots did not possess enough definition to clearly identify whether ice propagation stopped at the base of the bud (Workmaster et al., 1999). In order to understand the factors driving variations in the cold hardiness of the terminal buds of this species during acclimation and deacclimation, it is important to focus on the anatomical changes that may contribute to the presence of an ice barrier. Such studies would contribute to assessing the consequences of environmental stresses over this species.

This study aimed to determine the presence and nature of an ice barrier in *V. macrocarpon* shoots and to define its role in the development of the freezing tolerance of terminal buds. To address this objective, we measured bud freezing tolerance by controlled freezing tests and ice nucleation distribution with thermal videography over the stem and bud at three points during dormancy: early fall in September, winter in January, and spring in May. In addition, we focused on two zones of the *V. macrocarpon* shoot, the bud base and the stem, using histological and cytological techniques. Finally, we assessed the presence of pectins in these zones using antibodies that target two types of homogalacturonan.

## Materials and Methods

### Plant Material

Terminal shoots of *V. macrocarpon*, cultivar HyRed, were collected from a commercial farm located in Nekoosa, Wisconsin on three dates: September 22, 2018, after terminal bud formation; January 10, 2019, when buds were endodormant; and May 10, 2019, just before bud swell. At each sampling date, handfuls of shoots were collected and placed in zippered bags and transported to the laboratory on ice. Sampling replicates were obtained by randomly collecting shoots from each of one-third sections of a production bed that was approximately 50 m × 250 m. Samples were processed within 3 h after field sampling. Shoots were sorted in the laboratory and those containing a single medium-size terminal bud (1–2 mm diameter) were selected for all evaluations.

### Controlled Freezing Test

Controlled freezing tests (CFT) were performed on the same days as sampling, based on the methodology described by Villouta et al. (2020). Tests were performed in a Tenney Model T2C programmable freezing chamber (Thermal Product Solutions, New Columbia, PA, United States). Temperatures inside the chamber were monitored with two copper-constantan (Type T) thermocouples (22 AWG) placed inside 50 ml capped plastic centrifuge tubes. Thermocouples were connected to a Keithley 2700-DAQ-40 multimeter data acquisition system (Keithley Instruments, Cleveland, OH). Temperature readings were recorded at a 6-s interval with a Keithley add-in in Excel (Microsoft Corp., Redmond, WA).

Shoot preparation consisted of a rinse with tap water, followed by cutting underwater to 8 cm, and blotting dry with paper towels. Groups of five shoots, wrapped at their collective base with a small piece of moist paper towel, were placed in 50 ml capped plastic centrifuge tubes. Three replicate tubes were used for each test temperature. A set of three replicate tubes served as an unfrozen control and were kept on ice. The freezing chamber temperature program for each CFT started with a thermal equilibrium step at 1°C, followed by a temperature decrease to −1°C at a rate of 1°C/h. After 30 min at −1°C, the racks of tubes were firmly tapped to elicit ice nucleation, after which the temperature was held at −1°C for an additional hour. Afterward, three different freezing rates were used: 1, 2, and 4°C/h, until reaching −6°C, −12°C, and the lowest evaluated temperatures, respectively. For each CFT, nine test temperatures were used, spanning between 0 and −24°C in September 2018, 0 to −50°C in January 2019, and from 0 to −26°C in May 2019. Once each set of tubes was removed from the freezing chamber, tubes were kept on ice for 12 h, then transferred to a refrigerator at 4°C, and maintained in the dark for 3 days to allow any potential injury recovery. Next, tubes were kept in low light at room temperature for 24 h to facilitate damage expression of tissue browning.

### Freezing Stress Damage Evaluation

All 150 shoots from each CFT were assessed for freezing damage. Damage evaluation of dissected buds was performed according to the methodology described by Villouta et al. (2020). Samples were assessed using an Olympus SZX12 dissection microscope with 10X oculars and 1X objective (Olympus Optical Company, Tokyo, Japan) connected to a Canon EOS Rebel T6i digital camera (Canon United StatesA, Inc., Melville, NY). For damage assessment, buds were exposed by removing the nearest leaves. Buds were excised, retaining approximately 5 mm of stem. The sample was longitudinally dissected, cutting with one side of a double-edged razor blade. Damage evaluation was organized by bud structure: shoot apical meristem (SAM), flower primordia, bud axis, attached stem, and bud scales. Freezing damage severity was scored for each evaluated structure by the proportion of oxidative browning (Larsen, 2009) and water-soaked appearance as described by Villouta et al. (2020). A scale from 0 to 3 was used, with 0 representing no damage, 1 representing damage up to one third of the area of the evaluated structure, 2 representing damage up to two thirds of the area of the evaluated structure, and 3 representing complete damage of the structure.

### Sample Preparation for Microscopy

From each shoot collection sampling date, four buds were randomly selected for histological and cytological evaluations. The fixation, dehydration, embedding, and cutting of the samples were performed as described by Bolivar-Medina et al. (2018). After every sampling date, leaves were removed and buds were excised, retaining 5-mm of stem. For fixation, samples were immersed in a 4% glutaraldehyde solution (Sigma-Aldrich, St Louis, MO, United States) overnight at 4°C, and then rinsed four times in a 0.05 M potassium phosphate buffer. Samples were dehydrated through a graded ethanol series from 30 to 100% concentration and embedded by gradual replacement into medium-grade LR White resin (Ted Pella, Inc., Redding, CA, United States). Resin polymerization was performed by heating samples at 60°C for 28 h, followed by sample mounting on stubs for later longitudinal or transversal sectioning (2 μm thickness) on a Sorvall MT-2 ultramicrotome (Ivan Sorvall, Norwalk, CT, United States). Thin sections were placed on Fisher Probe-On-Plus slides (Thermo Fisher Scientific, Waltham, MA, United States) for staining.

### Methylene Blue and Basic Fuchsin Staining

**Figure 1:**
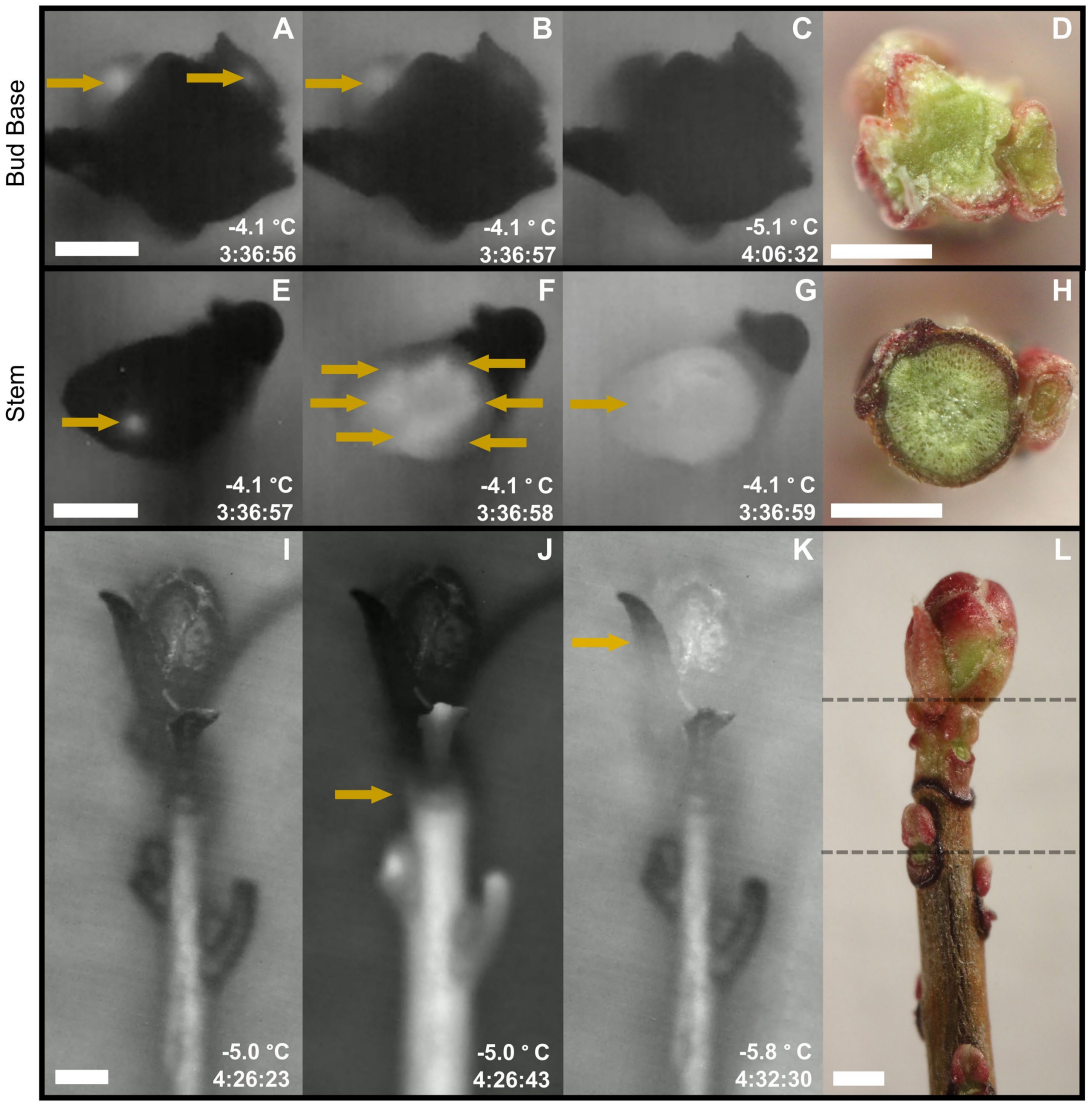
Longitudinal and transversal high-definition thermal recordings of *Vaccinium macrocarpon* freezing patterns in freezing tolerant terminal buds. Time stamps represents the time elapse under the freezing treatment, only comparable under the same sampling date. Arrows highlight ice formation propagation. **(A-C)** Thermal images of transversal sections at the base of the bud. This sequence of images shows ice propagation from the base of the stem toward the exposed cut, where ice did not reach the surface. **(D)** Fresh cut of a transverse section made at the base of the bud. **(E–G)** Thermal images of transversal sections of the stem. This sequence of images shows ice propagation from the base of the stem toward the exposed cut, where ice reaches the surface. Ice formation reaches the surface first, most likely, through vascular tissues. **(H)** Fresh cut of a transversal section made at the stem zone. **(I–K)** Thermal images of shoots partially defoliated. This sequence of images shows ice propagation from the base of the stem toward the bud, where ice propagation stops at the base of the bud **(J)**. Minutes later, ice formation is initiated in the bud **(K)**. **(L)** Fresh shoot manually defoliated depicting location of bud base (upper dashed line) and stem zone (lower dashed line). Scale bar = 0.5 mm.

Section staining was performed according to the protocol developed by Humphrey and Pittman (1974). Sections were embedded and cut as previously described and mounted on microscope slides. Evaluations focused on the proximal bud base and distal stem portions (Figure 1L). Sections were submerged in a methylene blue-azure II solution for 20 min at 65°C, followed by two rinses of distilled water to remove excess dye. Sections were blotted dry with paper towels and slides were incubated for 15 s with 0.05% basic fuchsin stain in 2.5% ethanol and rinsed with distilled water. Slides were dried at room temperature and coverslips annealed with Cytoseal 60 (Thomas Scientific, Riverdale, NJ, United States). Evaluations were performed using a bright-field Olympus BX50 microscope (Olympus Optical Company) connected to a Canon EOS Rebel T6i digital camera (Canon United States, Inc., Melville, NY). The resulting colors are cytoplasm and chloroplasts staining gray-blue, primary cell walls blue, and secondary cell walls with high cellulose content will stain pink.

### Histological and Cytological Evaluation

For histological and cytological analysis, evaluations were focused on the zones of the bud base and the stem (Figure 1). Areas of 25,000 μm^2^ for xylem and 15,000 μm^2^ for pith were anatomically characterized in each zone for each of the four repetitions and sampling date. Xylem conduits were independently evaluated according to both types present in the *V. macrocarpon* stem, tracheids and vessel elements (Addoms and Mounce, 1931) and just tracheids at the bud base. The parameters evaluated for xylem conduits and pith cells were: inner diameter (tracheids, and vessel elements), cell wall thickness, and the ratio of cell wall thickness to inner cell diameter. The arithmetic inner diameter was obtained by calculating the diameter of a circle with the same lumen area. Specifically for xylem conduits, the hydraulic weighted mean diameter was calculated to estimate the vessels’ contribution to hydraulic conductance. For each conduit, the diameter (*d*) was calculated from the lumen area. The mean was calculated as: ∑*d*^5^/∑*d*^4^ (Kolb and Sperry, 1999). In addition, the number of tracheids and vessel elements, whole xylem area, percentage of pith intercellular spaces, whole pith diameter, and stem diameter were measured. The total conducting xylem area and the total number of xylem conduits were estimated from the values obtained from the assessed 25,000 μm^2^ area and extrapolated to the entire xylem area. Images were analyzed with image software ImageJ (National Institutes of Health; Schneider et al., 2012).

### Pectin Immunolocalization

Two antibodies were used to determine the presence of different states of HG: rat monoclonal antibodies LM19 against de-methyl-esterified HG and LM20 against methyl-esterified HG (PlantProbes, University of Leeds, England; Verhertbruggen et al., 2009). Evaluations focused on the proximal bud base and distal stem portions (Figure 1L). For immunofluorescence labeling, 3 μm thick sections of LR White-embedded material were used, obtained as described above. Sections were blocked in 3% bovine serum albumin (BSA) in 1x phosphate-buffered saline (PBS; pH 7.2) for 30 min at room temperature to mask non-specific binding sites. Then, samples were incubated with the primary antibody (1:5 in PBS) for 2 h at room temperature. Samples were then washed twice with a high salt PBS wash solution for 10 min each time, followed by a brief rinse of PBS/BSA blocking solution. Next, sections were incubated with the secondary antibody (goat anti-rat-IgM Cross-Adsorbed) linked to DyLight 594 (Invitrogen, Thermo Fisher Scientific) diluted 1:100 in PBS for 1 h at room temperature. Samples were then washed twice in PBS for 15 min each time and rinsed with distilled water for 15 min. Section mounting was done with Citifluor AF1 (EMS, United States). Control sections followed the same staining protocol but excluding the primary antibody. Samples were examined using a confocal microscope Zeiss LSM-710 (Carl Zeiss, Oberkochen, Germany) with 10x oculars and 10x and 20x objectives. Samples were excited with a 594 nm laser line, and emission was collected between 600 and 635 nm. Image analysis was performed with ImageJ to evaluate relative fluorescence intensity, with values obtained from controls subtracted from fully stained sections.

### Thermal Videography

For each sampling date, 90 shoots were randomly chosen among the previously sorted samples collected from the field, wrapped in moist paper towels, placed in 0.65 L zip bags, placed inside a Styrofoam box with cold packs, and shipped overnight for thermal videography studies in the laboratory of David Livingston (United States Department of Agriculture–Agricultural Research Service, North Carolina State University). Samples were stored at 3°C in darkness upon receipt in North Carolina.

For thermal videography, ice nucleation patterns were monitored with a digital infrared camera FLIR T620 (FLIR Systems, Wilsonville, OR) with a 45° lens with 0.05°C thermal sensitivity. For each sampling date, three videos were recorded using eight shoots each. The camera was placed inside a freezer and connected to a desktop computer *via* a USB connection, and videos were recorded using FLIR “Research IR” software (FLIR Systems, Wilsonville, OR). Shoots were stripped of most leaves to expose the terminal bud, while the base of the shoot was inserted into a moist soil mix [Farfard 4P soil mix (Sungro Horticulture, Agawam, MA)]. This soil spontaneously freezes between −0.5 and −2°C, so no inoculum was added to induce ice nucleation. Samples were placed against a black velvet cloth to provide a consistent background and contrast with the plant tissue. Freezing temperatures began at 0°C and ramped to −10°C at 1°C/h. For high-definition thermal recordings, a digital infrared camera FLIR SC8303 (FLIR Systems, Wilsonville, OR) with a close-up lens 3–5 μm, 1X microscope f/4HD, and with <25 mK thermal sensitivity was used, following the same protocol as previously described.

### Statistical Analysis

Results were compared within each evaluated zone and compared across sampling dates. Differences in mean values were tested through one-way ANOVA and Tukey test (*p* ≤ 0.05). When ANOVA assumptions were not satisfied, the non-parametric Kruskal-Wallis and subsequent Dunn test were performed. All statistical analyses were performed using R software (ver. 3.5.1, R Foundation for Statistical Computing).

## Results

### Controlled Freezing Test

**Figure 2:**
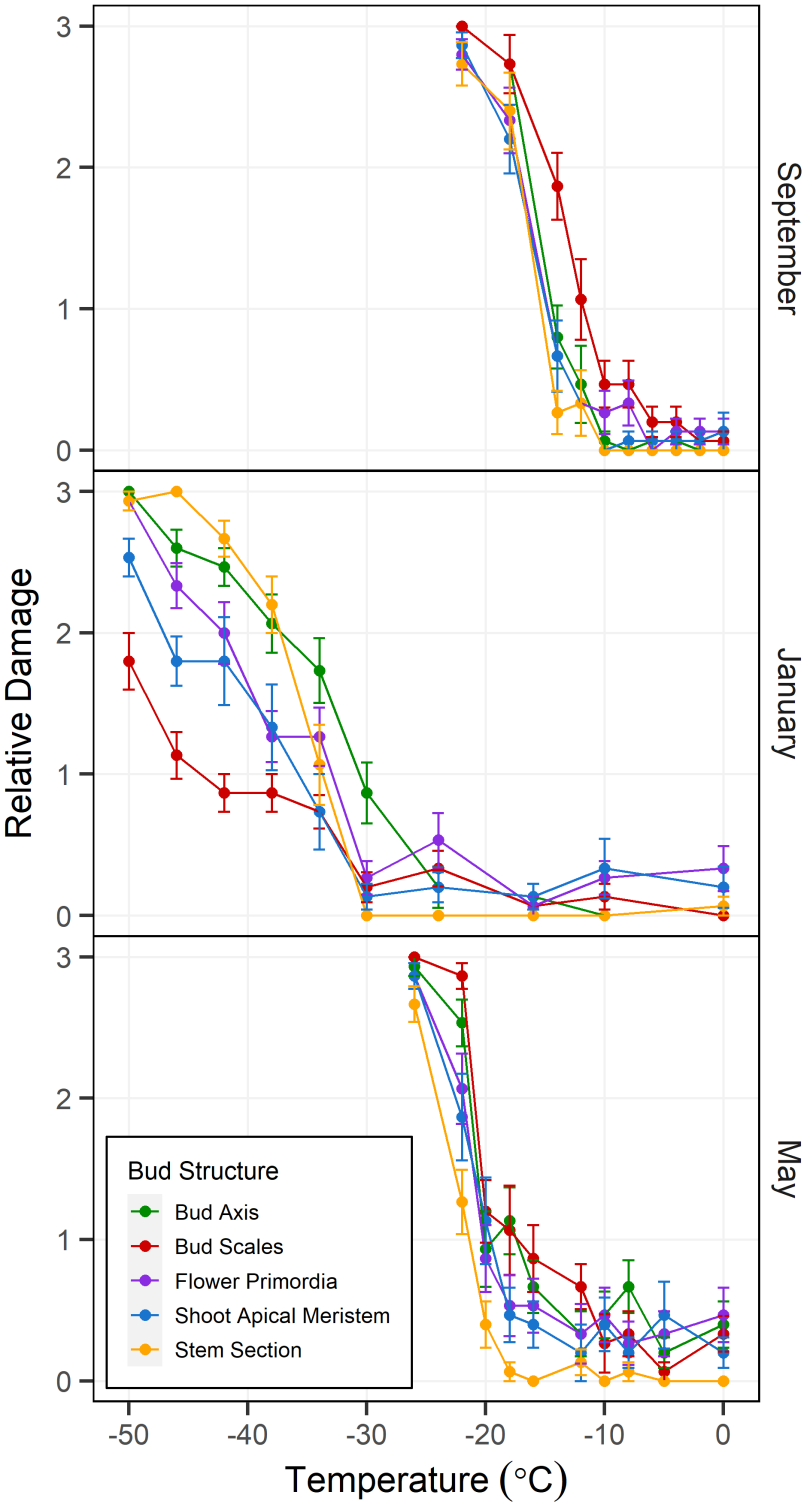
Controlled freezing test-incurred damage according to bud structure in *Vaccinium macrocarpon* terminal buds. Plant material was sampled in September 2018 and January and May 2019 from a commercial farm in Nekoosa, WI. Damage scoring was performed according to a discrete scale of damage from 0 to 3, where 0 represents no damage and 3 maximum damage. Damage was considered as the area with brown and water-soaked tissues. Each point was obtained from the average damage on each structure (*n* = 15). Vertical bars represent SE from the mean.

Freezing tolerance of buds at each date was determined by the severity of browning and water soaked appearance in each of the evaluated structures: SAM, flower primordia, bud axis, bud scales, and stem section. In September, damage ratings remained at levels below 0.5 until reaching −10°C, where a abrupt increase in damage was detected (Figure 2), with maximum damage occurring at −24°C. In general, all structures exhibited similar patterns of damage, with the exception of the bud scales showing higher severity in comparison to the other evaluated bud sections. For samples collected in January, damage remained at levels below 0.5 until −30°C, after which damage severity progressively increased without all the structures experiencing maximum damage (Figure 2). When comparing structures for the maximum damage reached at the lowest test temperature (−50°C), the SAM and bud scales had lower levels, while flower primordia, bud axis and stem section reached maximal levels of damage. For the last sampling date (May), damage levels increased slowly between −10 and −20°C, followed by a rapid increase until reaching maximal damage at −26°C. In general, all the structures exhibited a similar pattern of damage as temperature decreased, with the exception of the stem section which, on average, had the lowest incidence of damage at most of the test temperatures (Figure 2).

### Thermal Videography

**Figure 3:**
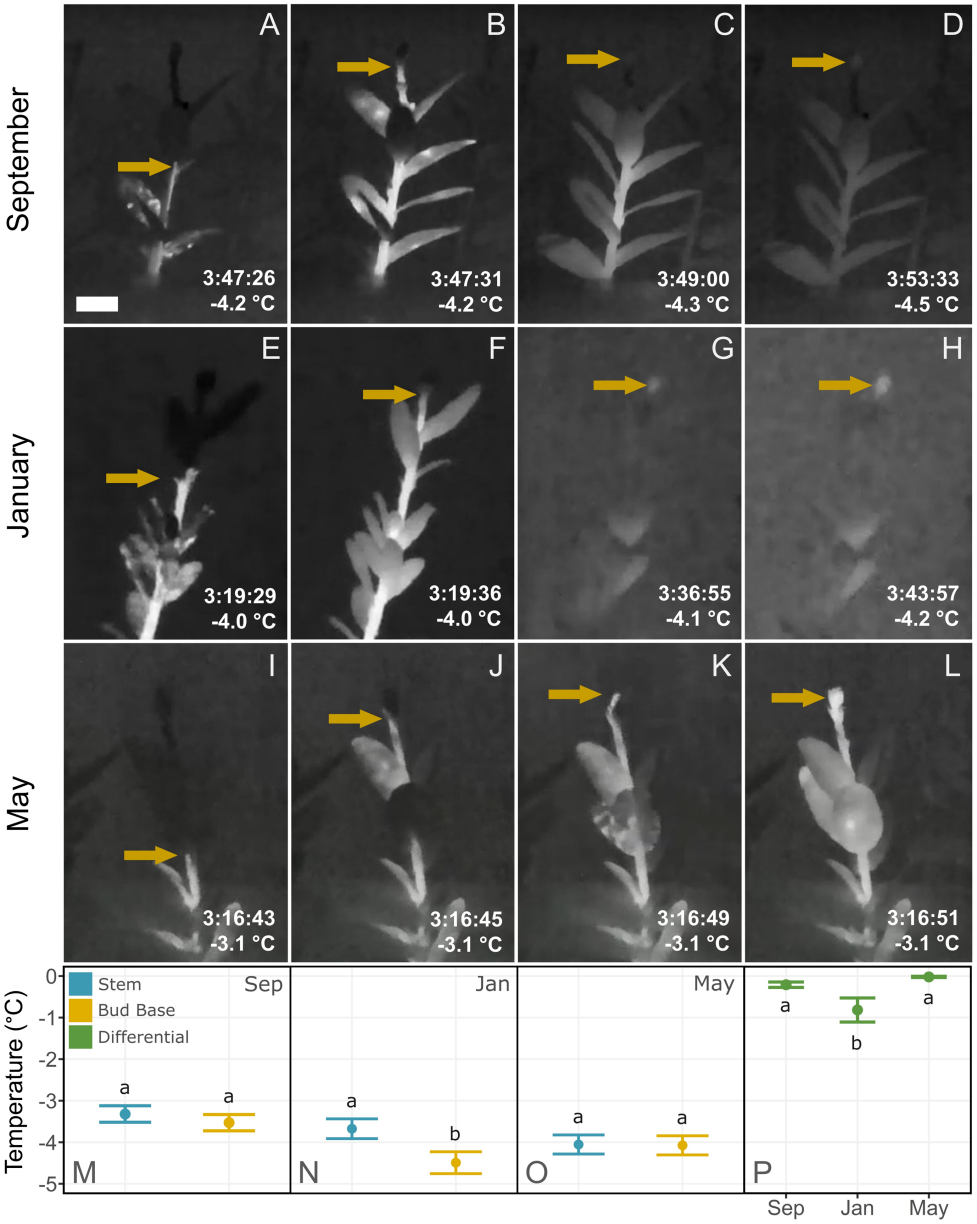
Thermal recordings of freezing patterns in *Vaccinium macrocarpon* shoots. Plant material was sampled in September 2018 and January and May 2019 from a commercial farm in Nekoosa, WI. Time stamps represents the time elapse under the freezing treatment, only comparable under the same structure. Arrows highlight ice formation propagation. **(A–D)** Ice propagation in a shoot sampled in September 2018. **(B,C)** Arrow indicates ice propagation stops at the base of the bud. **(D)** Shortly after, bud freezing resumes, indicated by the arrow. **(E–H)** Ice propagation in a shoot sampled in January 2019. **(F,G)** Arrow indicates where ice propagation stops at the base of the bud. **(H)** Minutes later, bud freezing resumes, signified by the arrow. **(I–L)** Ice propagation in a shoot sampled in May 2019. **(J,K)** Arrows indicate where ice propagation progresses through the stem. **(G,H)** Ice propagation progresses uninterrupted into the bud. **(M–O)** Average temperature of initiation of freezing in the stem or bud for **(M)** September 2018, **(N)** January 2019, and **(O)** May 2019. **(P)** Differences between the freezing temperature of stem and bud at each sampling date. Lower case letters represent significant difference among the means in each plot. Scale bar = 5 mm.

In most shoots, ice propagated acropetally from the base of the stem towards the terminal bud, and an ice nucleation event occurred in the stem shortly after the moist substrate froze. Shoots sampled in September and January exhibited a temporal barrier-like zone at the base of buds where the propagation of ice from the stem was impeded (Figures 3B,F). In contrast, in May, ice propagated uninterrupted through the base of the bud into the internal bud structures (Figures 3J–L). The thermal recordings of two different transverse sections showed that ice propagation did not reach the bud base (Figures 1A–C), but did reach the cut surface at the stem zone (Figures 1E–G). In the latter, it was possible to observe ice propagation first through the xylem and then into the surrounding tissues (Figure 1F).

In samples collected in September, bud freezing began shortly after the stem froze between −3.1 and −4.7°C on average, while freezing of the bud occurred slowly, lasting for several minutes (Figures 3C,D). In samples collected in January, both freeze events also occurred differently, however the bud froze on average at a temperature only 0.8°C lower than the stem, which had an average freezing temperature of −3.7°C. As in the September samples, this bud froze slowly, lasting for several minutes (Figures 3G,H). In the samples collected in May, only one freeze event was detected, with ice propagating unhindered from the stem into the bud interior (Figures 3K,L), occurring on average at −4.1°C. This bud freezing occurred rapidly and with the same relative intensity (heat release) as in the stem. The temperature difference between the stem and bud freeze events was only statistically significant in the shoots collected in January (Figure 3P).

### Histological and Cytological Evaluation

**Figure 4:**
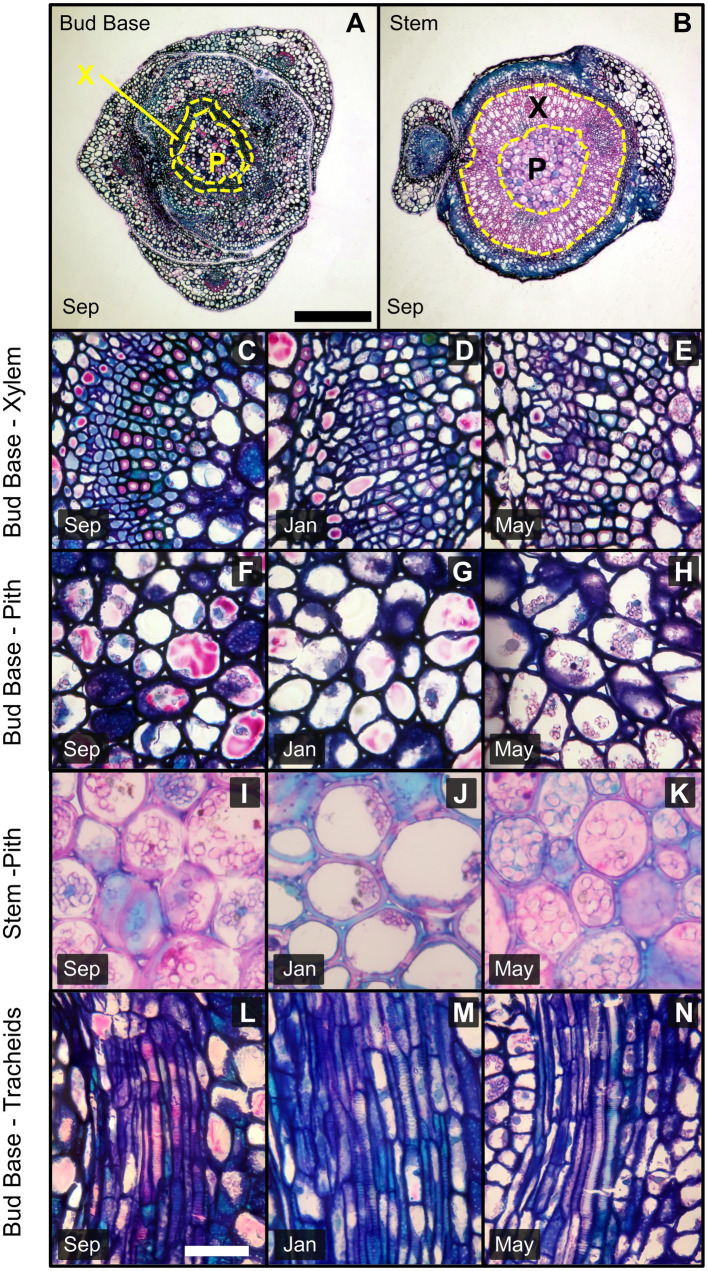
Images of transverse and longitudinal sections of the bud base and stem of *Vaccinium macrocarpon* shoots. Sections were stained with methylene FIGURE 4blue and basic fuchsin; dark blue indicates primary cell wall and pink/magenta secondary cell wall with high cellulose content. **(A)** Transverse section in the bud base and **(B)** in the stem zone both indicating xylem and pith area. **(C–E)** Transverse sections of vascular tissues in the bud base for **(C)** September, **(D)** January, and **(E)** May. **(F–H)** Transverse sections of pith tissue in the bud base for **(F)** September, **(G)** January, and **(H)** May. **(I–K)** Transverse sections of pith tissue in the stem for **(I)** September, **(J)** January, and **(K)** May. **(L–N)** Longitudinal sections of the bud base showing tracheids for **(L)** September, **(M)** January, and **(N)** May. X: Xylem, P: Pith. Scale bars **(A,B)** = 0.5 mm, **(C–N)** = 25 μm.

From the transverse sections, it was possible to identify the pith surrounded by vascular tissue (Figures 4A,B). Pith cells in the stem zone had secondary cell walls. However, distally to the bud base, pith cells had primary cell walls only (Figures 4F–H). For vascular tissues, specifically xylem in the stem zone, tracheids and vessel elements were observed. In contrast, there was an absence of vessel elements at the bud base, while tracheids continued toward the bud axis (Figures 4I–K).

**Figure 5:**
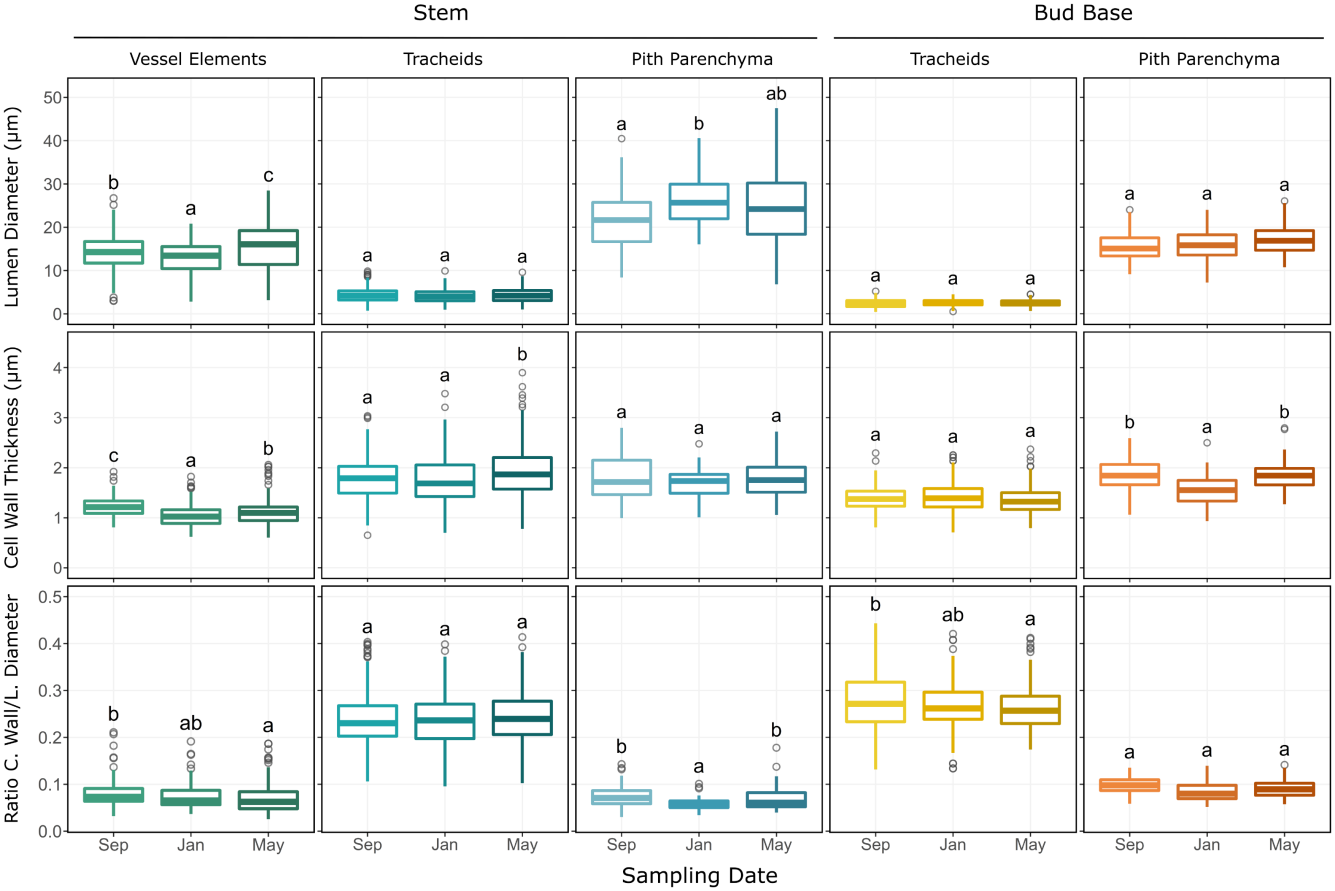
Histological parameters measured on transverse sections of *Vaccinium macrocarpon* terminal buds in the bud base and stem. Each plot depicts boxplots for each of the sampling dates. Each column represents a particular cell type or tissue, sub-grouped by stem and bud base. Rows represent the measured parameter. Whiskers represent the interquartile range 1.5-fold, while outliers are represented as gray rings. Lower case letters represent significant difference among the means in each plot.

Anatomical measurements of the bud base and stem zones along the shoot were used to assess seasonal differences. Measurements of xylem components for the stem (vessel elements and tracheids) and bud base (tracheids) did not reveal any seasonal differences for the hydraulic mean diameter (Table 1). The average inner diameter of stem vessel elements varied across sampling dates, decreasing from 14.3 μm in September to 13.0 μm in January, followed by an increase in May to 15.6 μm. The average cell wall thickness of vessel elements also varied among sampling dates, decreasing in January to 1.05 μm in comparison to 1.22 μm in September and 1.12 μm in May. Their average ratio of cell wall thickness to lumen diameter decreased from 0.078 to 0.070 μm from September to May samples. The average cell wall thickness of tracheids increased from 1.79 μm in September to 2.03 μm in May samples (Figure 5). The average ratio of cell wall thickness to lumen diameter of tracheids at the bud base also varied, decreasing from 0.277 in September to 0.267 in January, and 0.261 in May (Figure 5).

**Table 1:** Arithmetic mean diameter (AD) and hydraulically weighted mean diameter (HD) of xylem conduits at the bud base and stem zones of *Vaccinium macrocarpon*.

Xylem diameter (μm) ± SE by sampling date										
Cell type	Diam type	Sep			Jan			May		
Bud base tracheids	HD	3.15	±	0.28 a	4.04	±	0.07 a	3.41	±	0.22 a
	AD	2.38	±	0.05 a	2.54	±	0.05 a	2.52	±	0.75 a
Stem tracheids	HD	5.30	±	0.29 a	5.20	±	0.11 a	5.71	±	0.25 a
	AD	4.20	±	0.06 a	4.06	±	0.06 a	4.22	±	0.07 a
Stem vessel elements	HD	17.19	±	0.52 a	16.28	±	0.94 a	19.78	±	1.97 a
	AD	14.30	±	0.30 b	13.00	±	0.25 a	15.60	±	0.43 c

*Diameters were measured from the xylem conduits located within a radial section of 5,000 *μ*m^2^. Lower case letters represent significant difference among the means between sampling date*.

The average inner diameter of pith cells in the stem zone increased significantly among sampling dates, from 21.7 μm in September to 26.4 μm in January and was 24.1 μm in May; however no significant variations were observed in the bud base zone (Figure 5). Pith cell wall thickness decreased from 1.86 μm in September to 1.54 μm, in January, followed by an increase to 1.84 μm in May samples, but no variation in pith cell wall thickness was observed at the stem zone (Figure 5). The cell wall thickness to lumen diameter ratio decreased in the pith parenchyma at the stem from 0.074 in September to 0.059 in January, and increased to 0.070 in May samples, with no changes observed in the bud base (Figure 5).

**Figure 6:**
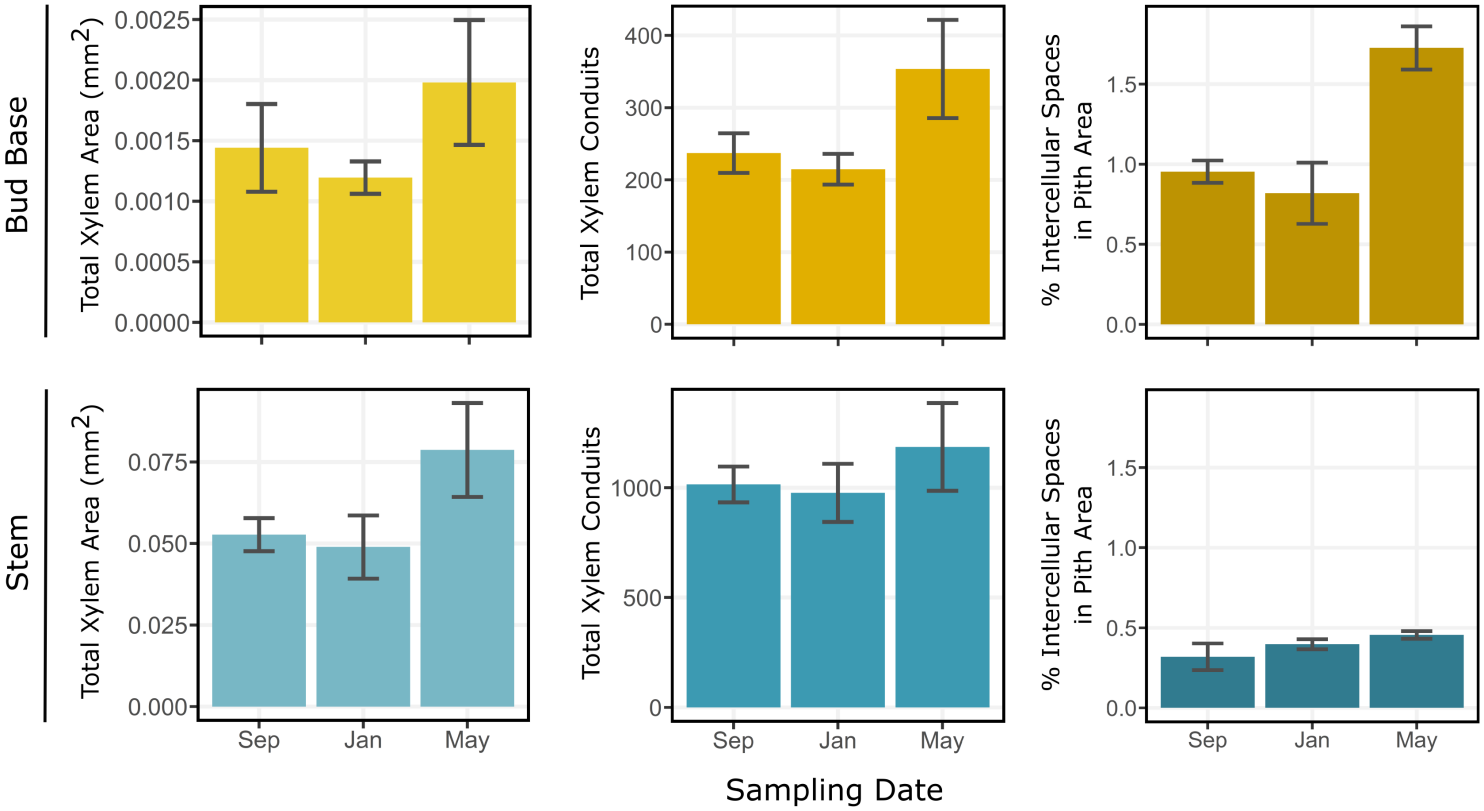
Estimations for total xylem area (mm^2^), total xylem conduits, and percentage of intercellular spaces in the pith of *Vaccinium macrocarpon* terminal buds in the bud base and stem. Rows represent bud base and stem. Columns represent the estimated parameter. Vertical bars represent SE of the mean.

Estimates of total xylem area, total xylem conduits, and percentage of intercellular spaces in the pith area generally increased from September to May sampling dates. Between January and May, the largest variations occurred in the bud base. Total xylem area increased from 0.0013 mm^2^ in January to 0.0018 mm^2^ in May samples (Figure 6). Total xylem conduits increased from an average of 198 units in January to 329 units in May, a 66% increase. The percentage of intercellular spaces more than doubled from January to May samples, from 0.7 to 1.8%. These parameters also varied in the stem zone, where the total xylem area increased from 0.052 mm^2^ in January to 0.077 mm^2^ in May samples. Total xylem conduits in the stem zone increased from an average of 1,045 units in September to 1,420 units in May samples, and the percentages of intercellular spaces were 0.3% in September and 0.4% in May samples (Figure 6).

### Pectin Immunolocalization

**Figure 7:**
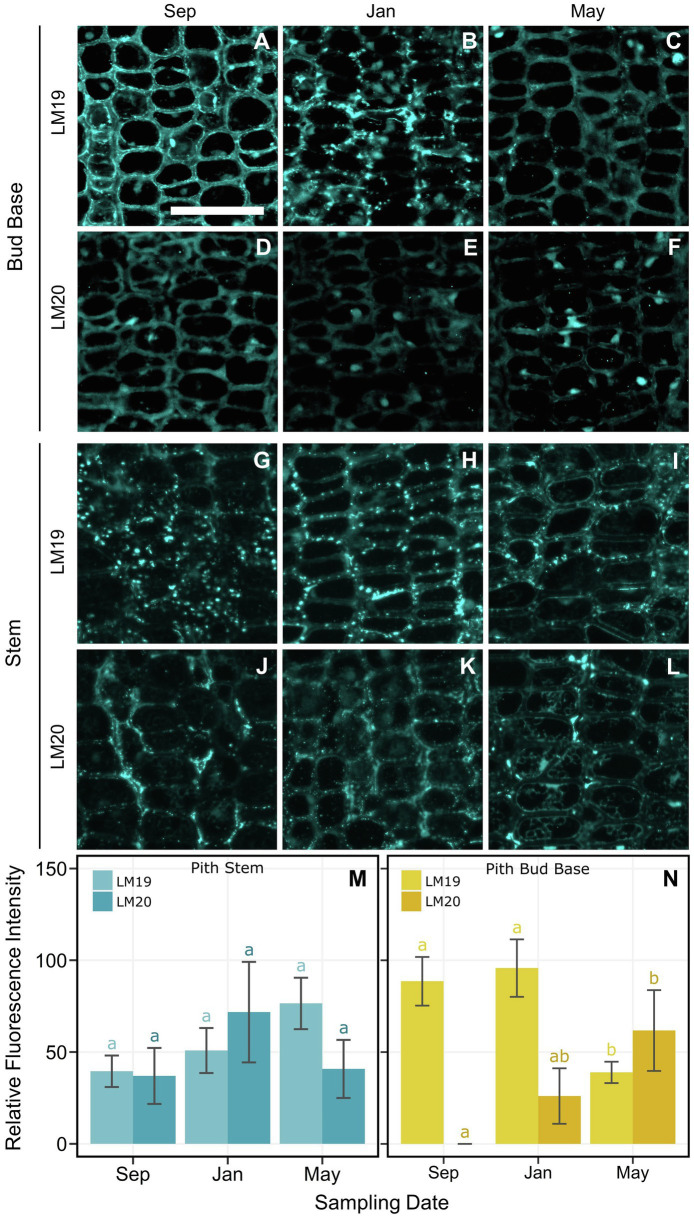
Indirect immunolocalization with antibodies **(A–C**,**G–I)** LM19 (de-methyl-esterified form) and **(D–F**,**J–L)** LM20 (methyl-esterified form), targeting homogalacturonan in pith cells of *Vaccinium macrocarpon*. **(A–L)** Columns represent sampling dates, rows represent sub-grouped evaluated zone, bud base or stem by antibody LM19 and LM20. **(M)** Bar plot for the average relative fluorescence intensity for pith cells in the stem. **(N)** Bar plot for the average relative fluorescence intensity for pith cells at the bud base. Lower case letters represent significant difference among the means of the same antibody and tissue in each plot. Vertical lines represent the SE of the mean. Scale bar = 50 μm.

**Figure 8:**
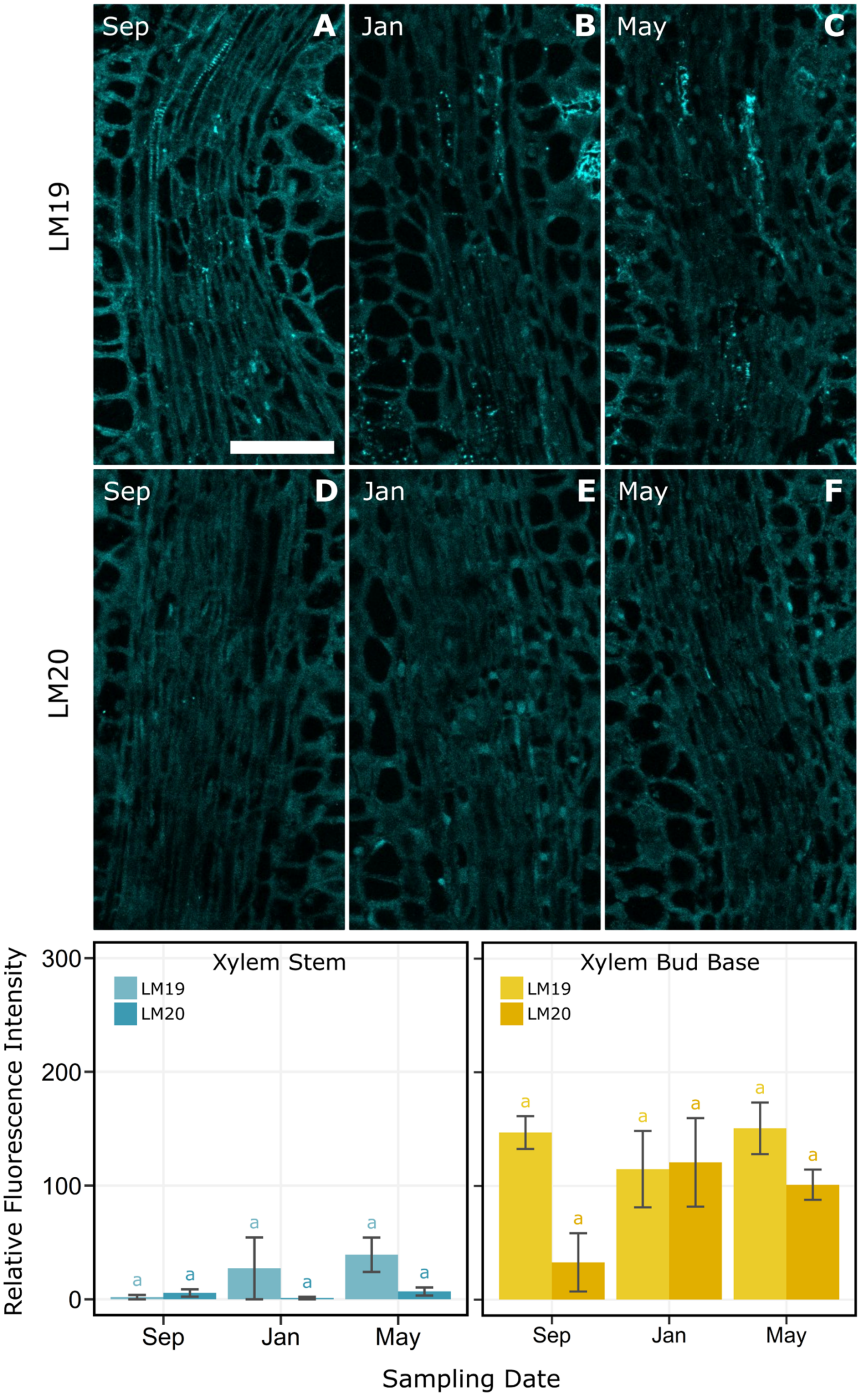
Indirect immunolocalization with antibodies **(A–C)** LM19 (de-methyl-esterified form) and **(D–F)** LM20 (methyl-esterified form) targeting homogalacturonan in xylem and xylem parenchyma cells of *Vaccinium macrocarpon*. Images of samples from **(A,D)** September, **(B,E)** January, and **(C,F)** May. Bar plot for the average relative fluorescence intensity for xylem and xylem parenchyma cells in the stem zone (bottom left). Bar plot for the average relative fluorescence intensity for xylem and xylem parenchyma cells in the bud base (bottom right). Lower case letters represent significant difference among the means of the same antibody and tissue in each plot. Vertical lines represent the SE of the mean. Scale bar = 50 μm.

Immunolocalization was used to measure the presence and location of the different methyl-esterified epitopes of homogalacturonan in cell walls of pith and xylem cells in the bud base and stem. In the pith of the bud base, LM20 was detected at low levels with a progressive increase across the three dates (Figure 7N). In contrast, LM19, which targets de-methyl-esterified HG, had high binding levels in September and January, which then decreased significantly in May (Figure 7N). LM19 was observed mainly at the cell walls, but the nature of it changed over time. In September, LM19 was homogenously distributed across cell walls (Figure 7A). By January, LM19 was visible as dots across the cell wall, which by May were a generally reduced (Figures 7B,C). At the stem zone, pith cell levels of LM19 and LM20 were not significantly different across sample dates (Figure 7M). However, the distribution of both antibodies differed across the cells. LM19 appeared as spots distributed through the cell wall (Figures 7G–I), while LM20 was present in the intercellular spaces, most often in the middle lamella (Figures 7J–L). Xylem at the base of the bud had higher levels of LM19 and LM20, compared with xylem in the stem, with no significant differences across dates (Figure 8).

## Discussion

This study aimed to identify the presence and nature of the ice barrier, as part of the freezing survival strategy by which *V. macrocarpon* terminal buds withstand below freezing temperatures. Our results confirm our hypothesis that *V. macrocarpon* buds are temporarily protected from exogenous ice nucleation *via* propagation from the stem through the presence of an ice barrier. We evaluated cold hardiness at three key phenological points to create a baseline of *V. macrocarpon* seasonal variations. *Via* thermal videography, we explored the localization of an ice barrier within the shoots. In addition, to assess how anatomical adaptation might have a role in impeding ice propagation from the stem, a combination of light and confocal microscopy were utilized to study the bud base and adjacent stem. While there were significant anatomical changes in the bud base which contributed to the formation of the barrier, the stem zone also changed to a smaller degree. These changes were mainly observed when comparing winter buds in January with spring buds in May. Changes in the nature of the ice barrier corresponded to variations in bud cold hardiness, where a relatively low level of hardiness was documented in September, followed by an increase in January, and a low level again in May.

### Ice Barrier Located at the Bud Base

The progression of freezing in *V. macrocarpon* shoots documented by thermal videography demonstrated that the ice barrier was located at the base of the bud. We observed a temporal interruption of ice propagation at the base of the bud during early fall (September) and winter (January), but not in spring (May; Figure 3). This delay was observed exogenously *via* IR thermography in intact shoots (Figure 3) and endogenously through transverse shoot sections (Figure 1). Barriers to ice propagation are a major component of the survival of buds during freezing conditions (Ashworth, 1984; Ashworth et al., 1992; Jones et al., 2000; Neuner et al., 2012, 2019; Endoh et al., 2014). A previous study surmised that *V. macrocarpon* terminal buds undergo freeze dehydration due to the existence of an ice barrier when exposed to low temperatures (Villouta et al., 2020). Workmaster and Palta (2006) also suggested that a barrier at the base of the bud would help explain the observed patterns of freezing damage in *V. macrocarpon* terminal buds. These studies are consistent with our findings of a significant delay in ice propagation from the stem into the bud (Figure 3).

### Nature of the Ice Barrier

To understand the nature of the ice barrier, we evaluated variations of methyl- and de-methyl-esterified HG in the pith and xylem of the bud base and the stem immediately below the bud base (Figure 1). The base of the bud, in comparison to the stem, had narrower and fewer xylem conduits, higher cell wall thickness to lumen diameter ratio in the tracheids, and a high content of de-methyl-esterified HG for the dates when the barrier was present. These anatomical features have been recognized previously as components of ice barriers in different species (George et al., 1974; Ashworth, 1982; Quamme et al., 1995; Wisniewski and Davis, 1995; Endoh et al., 2009; Neuner et al., 2012; Kuprian et al., 2014).

Because vascular tissues are the initial pathway for ice propagation (Neuner et al., 2010) an interruption of ice propagation implies an involvement of the tracheids at the bud base (Figure 4). It is possible that the formation of embolisms affected their functionality when exposed to freezing temperatures. This would cause a discontinuity in the water column from the stem into the bud, obstructing the propagation of ice through these tissues. However, causes of embolism are mostly related to drought stress or freeze–thaw cycles (Sperry and Tyree, 1988; Choat et al., 2016; Maruta et al., 2020), both unlikely conditions in our study conducted at a commercial farm, where vines were regularly irrigated by closely monitored soil water potential and the lack of freezing temperatures during the September sampling. It is also possible that during bud formation in the late summer tracheids are not fully functional and remain in this state through the winter. In addition, the lack of mature vascular conduits, specifically the presence of procambium strands toward the shoot apical meristem may also contribute as a barrier to ice propagation, as described in other species (Ashworth, 1982, 1984).

Demethylated pectins in the cell wall would limit water penetration, and could act as another type of barrier for ice propagation (Wisniewski et al., 1991). Pith cell walls in the bud base had a high content of de-methyl-esterified HG during the early fall and winter sampling dates (Figure 7). This form of HG is responsible for a decrease in the pore size of the cell wall matrix, reducing water movement (Wisniewski et al., 1991; Rajashekar and Lafta, 1996; Lee et al., 2017) due to Ca^2+^ crosslinking to HG when present in its de-methyl-esterified form Knox et al. (1990). Water, inside cell wall pores smaller than 5.2 nm, can supercool, limiting ice propagation (Ashworth and Abeles, 1984; Kuprian et al., 2016).

We also found a decrease in the cell wall thickness of the pith cells at the base of the bud for the January sampling date when buds were fully acclimated to cold temperatures. Previous studies have suggested that an increase in cell wall thickness would increase rigidity, thus avoiding cell collapse under dehydrative strain resulting from prolonged exposure to freezing temperatures (Huner et al., 1981; Rajashekar and Lafta, 1996). However, lower cell wall rigidity allows for the adjustment of cell osmotic potential necessary for cold acclimation (Pramsohler and Neuner, 2013). In *Malus domestica* Borkh. the bark tissue around the buds reached lower osmotic potentials than the tissues inside the bud during midwinter. In order to avoid water migration from the meristematic tissues inside the bud toward the bark and stem, the bud cells increase their cell wall elasticity to adjust their water potential (Pramsohler and Neuner, 2013). At the same time, changes in cell wall thickness have been reported to partially explain changes in cell elasticity, where a second factor is the cell wall composition (Zimmermann and Steudle, 1975). The role of active variation in cell wall elasticity as part of cold acclimation has been studied in conifers (Zwiazek et al., 2001) and desert shrubs (Scholz et al., 2012). In the case of *V. macrocarpon* cells, this idea would be in concordance with observed increases in plasma membrane unsaturated fatty acids during acclimation, resulting in increased membrane fluidity (Palta et al., 1993; Ndlovu, 2015).

In May, when ice progressed uninterrupted into the bud, indicative of the absence of the barrier, there was also an increase in the number of tracheids by 66% (Figure 6). The loss of barrier effectiveness due to re-establishment of connectivity between the bud and the stem has been widely documented (Ashworth, 1984; Ashworth et al., 1992; Julian et al., 2007; Neuner et al., 2012; Pramsohler and Neuner, 2013). An increase in the size of intercellular spaces, as we observed in May (Figure 6), has also been attributed with the disabling of barriers to ice propagation (Ashworth, 1984; Quamme et al., 1995; Jones et al., 2000).

### Anatomical and Metabolic Changes Contribute to Cold Hardiness Variations

**Figure 9:**
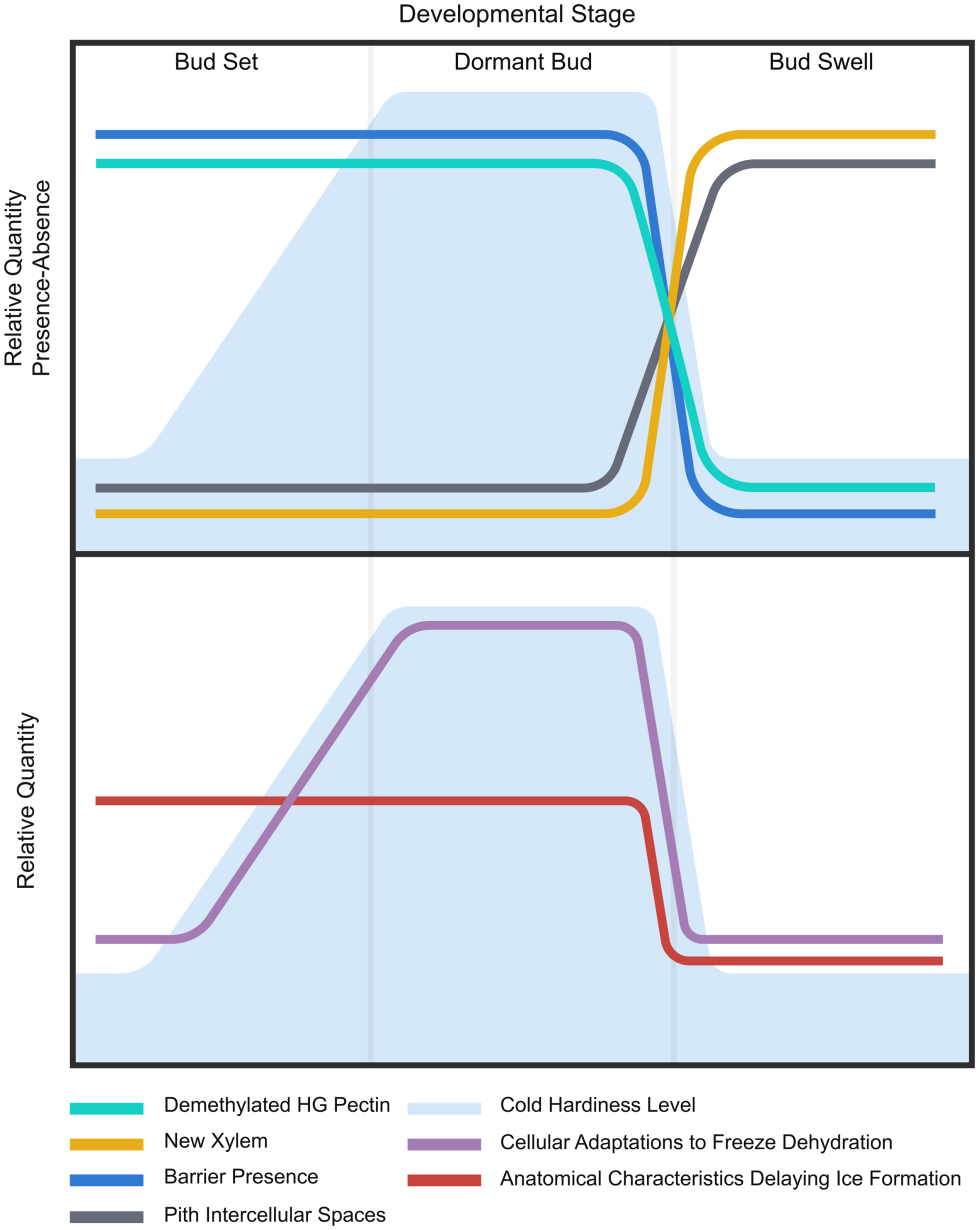
Summary of results and proposed hypothesis. (Upper) Lines depict general seasonal trends of evaluated elements considered part of the changes in cold hardiness in *Vaccinium macrocarpon* terminal buds. Background depicts variations in freezing tolerance. (Lower) Lines represent the proposed two levels of adaptations at anatomical and metabolic/cellular levels. Background depicts variations in freezing tolerance.

The development of cold hardiness in *V. macrocarpon* terminal buds apparently results from both anatomical and metabolic adaptations, although these do not necessarily occur concurrently (Figure 9). Adaptations at the anatomical level were associated with the lack of functionality of tracheids at the base of the bud and the reduced extracellular spaces in the pith in September and January. These two anatomical features are present since the formation of the bud in late summer and only change (allowing ice progression) when buds lose cold hardiness before resuming growth in spring. Metabolic adaptations would include: increases in nonstructural carbohydrates in response to low temperatures, such as sucrose, fructose, glucose (Ndlovu, 2015), raffinose, and stachyose (Deslauriers et al., 2021), as well as changes in lipid saturation in the plasma membrane (Palta et al., 1993; Ndlovu, 2015). These adjustments that most likely contributed to an increase in freezing tolerance in January, would begin during fall, reaching a maximum in January, and decrease again by May. However, the presence of an ice barrier in both the September and January sampling dates was not enough to develop freezing tolerance (Figure 2). Adaptations at the anatomical and metabolic levels would overlap in January, resulting in the maximum increase in freezing tolerance.

A potential explanation of the difference in the timing of these two acclimation factors could be associated with differences in the temperature thresholds that trigger acclimation and deacclimation patterns and their energetic costs. The anatomical component of the ice barrier, present since bud set, is of a constitutive nature, rather than an environmental response triggered by cold temperature, such as most cold acclimation responses (Fowler and Thomashow, 2002; Browse and Lange, 2004; Ndlovu, 2015). On the other hand, metabolic changes would be responsible for the progressive increase in freezing tolerance (Villouta et al., 2020) as temperatures decrease during the fall and winter. The vascular disconnection between buds and the subtending stem tissue and its essential role in freezing tolerance and dormancy has been widely studied (Ashworth, 1984; Ashworth et al., 1992; Bartolini and Giorgelli, 1994; Xie et al., 2018). However, in our study, the presence of tracheids at the bud base suggests a form of vascular discontinuity or a lack of functionality as contributing to stopping ice propagation. According to Savage and Chuine (2021) remains unclear when the connection between the stem and the bud’s xylem becomes functional and not just present.

In the case of deacclimation, it is possible that the development of new xylem drives the fast rate of the loss of freezing tolerance seen in this species (Workmaster and Palta, 2006; Villouta et al., 2021). The reactivation of cambial cells is regulated by air temperature (Begum et al., 2012), and is responsible for developing new xylem conduits, re-establishing the connection between the stem and the bud (Aloni, 2001, 2013). It is understood that spring xylogenesis would occur once a temperature threshold is reached (Schmitt et al., 2004; Rossi et al., 2007). However, the specific temperature threshold required for the resumption of vascular tissue formation in *V. macrocarpon* remains unclear. Different tissues can respond differentially to deacclimation temperatures (Takeuchi and Kasuga, 2018; Villouta et al., 2020), such that metabolic deacclimation processes may respond to different temperature thresholds than xylogenesis.

It is also possible that the timing of xylogenesis is linked to the general phenology of *V. macrocarpon*, where its deacclimation timing is tied to other growth stages, such as bud break and bloom. Non-precocious species, such as *V. macrocarpon*, display new leaf formation before reproductive structures during spring, allocating fewer resources to the formation of reproductive structures within buds during the year before, as compared to precocious species, which bloom before displaying leaf growth (Savage, 2019). Therefore, to resume growth in the spring, non-precocious species must start xylogenesis and reach vessel maturation before flowering to supply required water and solutes (Savage, 2019). This requirement for early vessel development and maturation would impact the isolation of buds and their level of cold hardiness (Ashworth, 1984; Ashworth et al., 1992; Bartolini and Giorgelli, 1994; Xie et al., 2018), resulting in faster deacclimation rates, such as are observed in this species (Villouta et al., 2021).

Several questions arise from our results regarding the cues influencing the timing and rates of these processes. Potential future research testing if catabolic processes related to deacclimation are a requirement for xylogenesis to begin would provide further insights. Determining the timing of xylogenesis initiation in comparison to the beginning of deacclimation would help define if xylogenesis and biochemical deacclimation processes respond to the same environmental cues. Answering these questions will contribute to a better understanding of the timing and influence of the processes conferring freezing tolerance in terminal buds, as well as the impact on their phenology.

## Conclusion

Our study identified a potential ice barrier in *V. macrocarpon* and we have described several of its anatomical characteristics. This barrier is partially responsible for the seasonal variations in freezing tolerance experienced by this woody evergreen perennial vine. The presence of the barrier is most likely a constitutive response to the bud’s development. In contrast, the timing of the development of new xylem observed during deacclimation is driven by environmental conditions, namely air temperature. Ice barriers in buds of species undergoing freeze dehydration contribute to the effectiveness of this freezing survival strategy. Our results also give insights into how components of freezing tolerance could be differentiated, such as metabolic and anatomical adaptations and how they relate to environmental cues. Future studies could explore the temperature thresholds and timing for spring xylogenesis and xylem activity resumption to indicate the loss of freezing tolerance. Knowing the different components involved in the development of freezing tolerance can provide breeders with screening tools targeting the selection of freezing tolerant genotypes.

## Data Availability Statement

The raw data supporting the conclusions of this article will be made available by the authors, without undue reservation.

## Author Contributions

Design of the research and writing the manuscript were completed by AA, BW, and CV. Performance of the research was done by CV and DL. Data analysis, collection, or interpretation were performed by AA, BW, CV, and DL. All authors contributed to the article and approved the submitted version.

## Funding

This research is based upon work supported by the National Institute of Food and Agriculture, United States Department of Agriculture, through Hatch project 1009297, administered by the College of Agricultural and Life Sciences, University of Wisconsin–Madison, and by the Wisconsin Cranberry Research and Education Foundation.

## Conflict of Interest

The authors declare that the research was conducted in the absence of any commercial or financial relationships that could be construed as a potential conflict of interest.

## Publisher’s Note

All claims expressed in this article are solely those of the authors and do not necessarily represent those of their affiliated organizations, or those of the publisher, the editors and the reviewers. Any product that may be evaluated in this article, or claim that may be made by its manufacturer, is not guaranteed or endorsed by the publisher.
